# Neural tube defects as a cause of death among stillbirths, infants, and children younger than 5 years in sub-Saharan Africa and southeast Asia: an analysis of the CHAMPS network

**DOI:** 10.1016/S2214-109X(23)00191-2

**Published:** 2023-06-01

**Authors:** Lola Madrid, Kartavya J Vyas, Vijaya Kancherla, Haleluya Leulseged, Parminder S Suchdev, Quique Bassat, Samba O Sow, Shams El Arifeen, Shabir A Madhi, Dickens Onyango, Ikechukwu Ogbuanu, J Anthony G Scott, Dianna Blau, Inacio Mandomando, Adama M Keita, Emily S Gurley, Sana Mahtab, Victor Akelo, Sulaiman Sannoh, Yenenesh Tilahun, Rosauro Varo, Uma Onwuchekwa, Afruna Rahman, Yasmin Adam, Richard Omore, Sandra Lako, Elisio Xerinda, Kazi Munisul Islam, Amy Wise, Beth A Tippet-Barr, Erick Kaluma, Sara Ajanovic, Karen L Kotloff, Mohammad Zahid Hossain, Portia Mutevedzi, Milagritos D Tapia, Emily Rogena, Francis Moses, Cynthia G Whitney, Nega Assefa, CHAMPS Consortium, A.S.M. Nawshad Uddin Ahmed, Mahbubul Hoque, Mohammed Kamal, Mohammad Mosiur, Ferdousi Begum, Saria Tasnim, Meerjady Sabrina Flora, Farida Arjuman, Iqbal Ansary Khan, Tahmina Shirin, Mahbubur Rahman, Sanwarul Bari, Shahana Parveen, Farzana Islam, Mohammad Zahid Hossain, Kazi Munisul Islam, Mohammad Sabbir Ahmed, K Zaman, Mustafizur Rahman, Dilruba Ahmed, Md Atique Iqbal Chowdhury, Muntasir Alam, Kyu Han Lee, Ferdousi Islam, Joseph O Oundo, Fikremelekot Temesgen, Melisachew Mulatu Yeshi, Alexander M Ibrahim, Tadesse Gure, Yunus Edris, Addisu Alemu, Dadi Marami, Ephrem Lemma, Ayantu Mekonnen, Henok Wale, Tseyon Tesfaye, Haleluya Leulseged, Tadesse Dufera, Anteneh Belachew, Fentabil Getnet, Surafel Fentaw, Yenework Acham, Stian MS Orlien, Mahlet Abayneh Gizaw, Emily Rogena, Florence Murila, Gunturu Revathi, Paul K Mitei, Magdalene Kuria, Jennifer R Verani, Aggrey Igunza, Peter Nyamthimba, Elizabeth Oele, Karen D Fairchild, Carol L Greene, Rima Koka, Ashka Mehta, Sharon M Tennant, J Kristie Johnson, Tatiana Keita, Adama Mamby Keita, Nana Kourouma, Uma U Onwuchekwa, Awa Traore, Doh Sanogo, Diakaridia Sidibe, Seydou Sissoko, Diakaridia Kone, Milton Kindcardett, Khátia Munguambe, Ariel Nhacolo, Tacilta Nhampossa, Pio Vitorino, Elisio Xerinda, Justina Bramugy, Celso Monjane, Sheila Nhachungue, Juan Carlos Hurtado, Maria Maixenchs, Clara Menéndez, Jaume Ordi, Natalia Rakislova, Marta Valente, Zara Manhique, Dercio Chitungo, Sibone Mocumbi, Carla Carrilho, Fabiola Fernandes, Rebecca Pass Philipsborn, Jeffrey P Koplan, Mischka Garel, Betsy Dewey, Shailesh Nair, Navit T Salzberg, Lucy Liu, Rebecca Alkis-Ramirez, Jana M Ritter, Sherif R Zaki, Joy Gary, Jonas M Winchell, Jacob Witherbee, Jessica L Waller, Ruby Fayorsey, Ronita Luke, Ima-Abasi Bassey, Dickens Kowuor, Foday Sesay, Baindu Kosia, Samuel Pratt, Carrie-Jo Cain, Solomon Samura, Fatima Solomon, Ashleigh Fritz, Noluthando Dludlu, Constance Ntuli, Richard Chawana, Karen Petersen, Sanjay G Lala, Sithembiso Velaphi, Jeannette Wadula, Martin Hale, Peter J Swart, Hennie Lombaard, Rahima Moosa, Gillian Sorour

**Affiliations:** a Department of Infectious Disease Epidemiology, London School of Hygiene & Tropical Medicine, London, UK; b College of Health and Medical Sciences, Haramaya University, Harar, Ethiopia; c Emory Global Health Institute, Emory University, Atlanta, GA, USA; d Rollins School of Public Health, Emory University, Atlanta, GA, USA; e Center for Global Health, US Centers for Disease Control and Prevention, Atlanta, Georgia, USA; f ISGlobal, Hospital Clínic, Universitat de Barcelona, Barcelona, Spain; g Centro de Investigação em Saúde de Manhiça (CISM), Maputo, Mozambique; h ICREA, Barcelona, Spain; i Paediatrics Department, Hospital Sant Joan de Déu, Universitat de Barcelona, Esplugues, Barcelona, Spain; j Consorcio de Investigación Biomédica en Red de Epidemiología y Salud Pública (CIBERESP), Madrid, Spain; k Centre pour le Développement des Vaccins, Ministère de la Santé, Bamako, Mali; l International Centre for Diarrhoeal Disease Research, Dhaka, Bangladesh; m South African Medical Research Council Vaccines and Infectious Diseases Analytics Research Unit, University of the Witwatersrand, Johannesburg, South Africa; n Kisumu County Department of Health, Kisumu, Kenya; o Ministry of Health and Sanitation, Freetown, Sierra Leone; p Crown Agents, Freetown, Sierra Leone; q KEMRI–Wellcome Trust Research Programme, Kilifi, Kenya; r Instituto Nacional de Saúde, Maputo, Mozambique; s Department of Epidemiology, Bloomberg School of Public Health, Johns Hopkins University, Baltimore, MD, USA; t US Centers for Disease Control and Prevention Kenya, Kisumu, Kenya; u Department of Pediatrics and Department of Medicine, Center for Vaccine Development and Global Health, University of Maryland School of Medicine, Baltimore, MD, USA; v Department of Pathology, School of Medicine, Jomo Kenyatta University of Agriculture and Technology, Nairobi, Kenya

## Abstract

**Background:**

Neural tube defects are common birth defects resulting in severe morbidity and mortality; they can largely be prevented with periconceptional maternal intake of folic acid. Understanding the occurrence of neural tube defects and their contribution to mortality in settings where their burden is highest could inform prevention and health-care policy. We aimed to estimate the mortality attributed to neural tube defects in seven countries in sub-Saharan Africa and southeast Asia.

**Methods:**

This analysis used data from the Child Health and Mortality Prevention Surveillance (CHAMPS) network and health and demographic surveillance systems from South Africa, Mozambique, Bangladesh, Kenya, Mali, Ethiopia, and Sierra Leone. All stillbirths and infants and children younger than 5 years who died, who were enrolled in CHAMPS, whose families consented to post-mortem minimally invasive tissue sampling (MITS) between Jan 1, 2017, and Dec 31, 2021, and who were assigned a cause of death by a determination of cause of death panel as of May 24, 2022, were included in this analysis, regardless the cause of death. MITS and advanced diagnostic methods were used to describe the frequency and characteristics of neural tube defects among eligible deaths, identify risk factors, and estimate the mortality fraction and mortality rate (per 10 000 births) by CHAMPS site.

**Findings:**

Causes of death were determined for 3232 stillbirths, infants, and children younger than 5 years, of whom 69 (2%) died with a neural tube defect. Most deaths with a neural tube defect were stillbirths (51 [74%]); 46 (67%) were neural tube defects incompatible with life (ie, anencephaly, craniorachischisis, or iniencephaly) and 22 (32%) were spina bifida. Deaths with a neural tube defect were more common in Ethiopia (adjusted odds ratio 8·09 [95% CI 2·84–23·02]), among female individuals (4·40 [2·44–7·93]), and among those whose mothers had no antenatal care (2·48 [1·12–5·51]). Ethiopia had the highest adjusted mortality fraction of deaths with neural tube defects (7·5% [6·7–8·4]) and the highest adjusted mortality rate attributed to neural tube defects (104·0 per 10 000 births [92·9–116·4]), 4–23 times greater than in any other site.

**Interpretation:**

CHAMPS identified neural tube defects, a largely preventable condition, as a common cause of death among stillbirths and neonatal deaths, especially in Ethiopia. Implementing interventions such as mandatory folic acid fortification could reduce mortality due to neural tube defects.

**Funding:**

Bill & Melinda Gates Foundation.

## Introduction

Neural tube defects include a group of serious birth defects characterised by incomplete neural tube closure between 21 and 28 days after conception.^1^ Spina bifida and anencephaly are the most prevalent neural tube defects affecting the brain and spine.

Inadequate folate status in women of reproductive age, substance abuse, and exposure to specific drugs early in pregnancy are well known risk factors for neural tube defects.^2^, ^3^, ^4^ The prevalence of folate insufficiency is more than 40% in most countries where data are available,^5^ including some African countries.^6^ Although 5–6 per 10 000 pregnancies appear not to be folate sensitive,^7^, ^8^ recommended interventions to prevent the majority of neural tube defects include supplementation and staple food fortification.^2^ However, preconception folic acid supplementation is only practised by 30% of mothers worldwide, similarly in sub-Saharan Africa, and by 21–46% of mothers in southeast Asia.^9^ As of 2021, about 70 countries have implemented mandatory food fortification with folic acid as a measure to prevent neural tube defects.^10^

**Research in context**

**Evidence before this study**

Neural tube defects are the most common severe central nervous system abnormality. They can be largely prevented with appropriate folic acid periconceptional intake. We undertook a systematic literature search of PubMed papers published in English between Jan 1, 2000, and Dec 31, 2021, that described mortality attributed to neural tube defects among stillbirths and children worldwide using the search terms and research strategy: “cause of death” AND (“congenital abnormalities” OR “neural tube defects” OR “anencephaly” OR “anencephaly” OR “encephalocele” OR “spina bifida” OR “craniorachischisis” OR “meningocele” OR “myelomeningocele”). The initial search yielded 1218 results and, through title and abstract review, we filtered these down to 23 studies that reported data on neural tube defects and mortality. Among these, only ten papers reported original data. Of those, six reported original data in children, five from high-income countries, only one from a middle-income country, Brazil, and none from a low-income or lower-middle-income country. The search did not identify any studies reporting original data on mortality attributed to neural tube defect in sub-Saharan Africa or southeast Asia. Modelled outcomes among children with neural tube defects in different UN subregions for the year 2015 reported a stillbirth prevalence of 4·5 and 2·7 per 10 000 livebirths in sub-Saharan Africa and southeast Asia, respectively. Modelled mortality attributable to neural tube defects under the age of 5 years was 0·8 and 0·6 per 10 000 livebirths in sub-Saharan Africa and southeast Asia, respectively. However, these estimates are not precise since comprehensive, population-based data on the mortality attributed to neural tube defects and associated factors are lacking in low-income and lower-middle-income countries due to the absence of robust birth defects surveillance systems, death registration, and linkages between them.

**Added value of this study**

The Child Health and Mortality Prevention Surveillance (CHAMPS) network works in 12 catchment areas in seven countries in sub-Saharan Africa and southeast Asia, most of which have ongoing health and demographic surveillance systems (HDSS). CHAMPS uses post-mortem minimally invasive tissue sampling to determine the cause of death by taking tiny tissue specimens, undertaking microbiological and histopathologic examination, and taking photographs for better visualisation of the cause of death when there is a visible birth defect. Detailed mortality surveillance at both facility and community levels, combined with clinical information, verbal autopsies, post-mortem photographs, and laboratory investigations are used to understand specific causes of death, such as neural tube defects. Nested in HDSS sites, the incidence and prevalence of fatal diseases can also be estimated. In this study, we present the specific mortality attributed to neural tube defects in the CHAMPS network and by site among more than 3000 stillbirths and child deaths reviewed, the epidemiological and maternal factors associated with this condition, and how these findings might be related to each country's national folic acid fortification policies and could inform prevention and health-care policy.

**Implications of all the available evidence**

Our comprehensive analyses of mortality fractions and all-cause total stillbirths and mortality in children younger than 5 years due to neural tube defects show that the mortality attributed to this condition in low-income and lower-middle-income countries is an important public health issue. Our findings provide evidence to support urgent action to prevent neural tube defects, especially in countries such as Ethiopia, where mortality with a neural tube defect was highest, to avoid neural tube defect-associated prenatal and postnatal mortality in the future.

The prevalence of neural tube defects varies globally and is disproportionately high in countries without mandatory food fortification programmes.^11^, ^12^ The global prevalence of spina bifida and anencephaly is estimated at 18·6 per 10 000 livebirths, with a regional prevalence of 13·1 per 10 000 livebirths in southeast Asia and 14·2 per 10 000 livebirths in sub-Saharan Africa.^12^ Fetuses with anencephaly die either during gestation or soon after birth.^4^, ^12^ Spina bifida can be disabling and has a high risk of resulting in stillbirth or death during the first 5 years of life, especially in low-income and lower-middle-income countries,^12^ and requires expensive, life-long medical and surgical care among those who survive.^4^ Literature on mortality among babies with neural tube defects and associated factors is scarce in low-income and lower-middle-income countries due to the absence of robust birth defect surveillance systems, death registration, and linkages between them.

The Child Health and Mortality Prevention Surveillance (CHAMPS) network was established to determine the causes of death among stillbirths, infants, and children younger than 5 years in South Africa, Mozambique, Bangladesh, Kenya, Mali, Ethiopia, and Sierra Leone by collecting post-mortem samples soon after death and conducting broad microbiological and histopathological testing. These results, along with data from clinical records, photographs, and verbal autopsy, were reviewed by trained multidisciplinary panels to attribute the most plausible causes and pathways leading to death.^13^

Only four (Bangladesh, Ethiopia, Kenya, and South Africa) of the seven countries where CHAMPS is operating have previously reported data on the prevalence of spina bifida and anencephaly, and these data are primarily based on hospital-based surveillance (appendix p 2). Among these four countries where CHAMPS is operating where prevalence data are available, Ethiopia has the highest prevalence of neural tube defects with an estimated pooled prevalence of 63 neural tube defects per 10 000 births.^14^ Prevalence in South Africa following mandatory folic acid fortification ranges between 8 and 10 per 10 000 births.^15^, ^16^

Our primary objective was to estimate the mortality attributed to neural tube defects in each CHAMPS site and, secondarily, to describe sociodemographic and maternal factors associated with neural tube defects and relate estimated neural tube defect burden to each country's national folic acid fortification policy.

## Methods

### Sites and mortality surveillance

This analysis used data from CHAMPS, whose site characteristics are described elsewhere.^13^ Briefly, CHAMPS was established in 12 catchment areas in seven countries in sub-Saharan Africa and southeast Asia: Baliakandi and Faridpur, Bangladesh; Harar, Kersa, and Haramaya, Ethiopia; Siaya and Manyatta, Kenya; Bamako, Mali; Manhiça and Quelimane, Mozambique; Makeni, Sierra Leone; and Soweto, South Africa.^13^ The CHAMPS sites began full mortality surveillance enrolment on the following dates: Dec 5, 2016 in Mozambique; Jan 16, 2017 in South Africa; May 4, 2017 Kenya; Aug 8, 2017 in Mali; Sept 20, 2017 in Bangladesh; Feb 4, 2019 in Ethiopia; and Feb 25, 2019 in Sierra Leone. All sites had estimated mortality greater than 50 deaths per 1000 livebirths in children younger than 5 years at the time of site selection (2015) and included both rural and urban areas.^13^

Site teams conducted mortality surveillance that captured facility and community deaths. Stillbirths or deaths among children younger than 5 years occurring among residents of the catchment area were eligible for study. For deaths captured within 24 h, written informed consent was obtained when possible for post-mortem procedures, including minimally invasive tissue sampling (MITS) of lung, brain, and liver, and samples of blood, cerebrospinal fluid cultures, and nasopharyngeal and rectal swabs. These samples were processed at local laboratories using standardised microbiological methods and multiplexed TaqMan (Thermo Fisher Scientific, Waltham, MA, USA) array cards. Tissue specimens were also reviewed by pathologists locally and at the US Centers for Disease Control and Prevention, using routine histopathology, special stains, and immunohistochemistry.^17^ Photographs to identify birth defects, anthropometric measurements, a verbal autopsy using the 2016 WHO verbal autopsy instrument,^18^ and antemortem clinical data were also collected. All data available for each death were reviewed by a determination of cause of death panel, which consisted of local experts in different disciplines (microbiologists, obstetricians, pathologists, paediatricians, and public health experts) who were trained to determine the chain of events leading to death using the tenth revision of the WHO International Classification of Diseases (ICD-10) and the WHO application of ICD-10 to deaths during the perinatal period.^19^, ^20^ Causes of death were categorised as immediate, underlying, or comorbid in the causal chain of events leading to death.

All stillbirths (no spontaneous breathing or movement at time of delivery and [1] weighing ≥1 kg or [2] estimated gestational age ≥28 weeks) and neonates (aged <28 days), infants (aged 28–364 days), and children (aged 1–5 years) who died, who were enrolled in CHAMPS, whose families consented to post-mortem sampling between Jan 1, 2017, and Dec 31, 2021, and who were assigned a cause of death by a determination of cause of death panel as of May 24, 2022, were included in this analysis, regardless of the cause of death.

Except Makeni in Sierra Leone, Quelimane in Mozambique, and Faridpur in Bangladesh, most sites carried out mortality surveillance within a health and demographic surveillance system (HDSS). These systems captured sociodemographic data, births, deaths, pregnancies, and migration episodes within a geographically defined area to estimate the size and structure of a population. HDSS platforms varied in maturity throughout the CHAMPS network;^21^ some were new during the study period and data were unavailable for this analysis. CHAMPS protocols and demographic and mortality surveillance methods have been previously described.^13^, ^22^ The CHAMPS protocol was approved by ethics committees in all sites and at Emory University, Atlanta, GA, USA.

### Statistical analysis

The minimum number of deaths that must have been enrolled in CHAMPS, had parental consent to MITS, and been assigned a cause of death to calculate the population-level cause-specific mortality fraction (CSMF) due to neural tube defects with 95% confidence and a 5% margin of error were 218 in Bangladesh, 249 in Ethiopia, 254 in Kenya, 183 in Mali, 292 in Mozambique, 196 in Sierra Leone, and 218 in South Africa.

Sampling was not performed because all eligible deaths in each target population were enumerated and characterised. Individuals were determined to have died with a neural tube defect if they were assigned one of the following ICD-10 codes anywhere in the causal chain (immediate, underlying, and comorbid causes): anencephaly (Q00.0), craniorachischisis (Q00.1), iniencephaly (Q00.2), encephalocele (Q01.0–Q01.9), and spina bifida (Q05.0–Q05.9). Institute for Health Metrics and Evaluation Global Burden of Disease categories were used to classify other causes of death in the causal chain. Child characteristics (site, age, sex, death location, year, season, and verbal autopsy cause of death) and maternal characteristics (age, religion, education, alcohol use, smoking status, and number of antenatal care visits) were described for all reviewed deaths. χ^2^ tests were done to compare the characteristics between deaths reviewed by determination of cause of death panels (ie, all deaths *vs* deaths with a neural tube defect) and to compare those who consented to MITS versus those who did not consent to MITS. Fisher's exact tests were performed if the expected value was less than one or fewer than five for at least 20% of the cell counts. A multivariable logistic regression analysis was performed to identify sociodemographic and maternal factors associated with deaths with a neural tube defect in the causal chain, using deaths from other causes as a comparison group. Exact logistic regression models were performed if the data were too sparse or skewed; rather than standard maximum-likelihood estimation, this method enumerates the exact distributions of sufficient statistics for the parameters of interest, conditional on the remaining parameters. Box-Tidwell tests were performed to check the linearity assumption between the log-odds of the outcome and each continuous independent variable. Adjusted odds ratios (ORs) and 95% CIs from reduced models in which all possible subsets of potential confounders had been removed were compared with those from the fully adjusted model; if the reduced model OR was within 10% of the OR for the fully adjusted model, there was evidence that the variable was not a confounder and was removed. Probability values less than 0·05 were considered significant.

Factors that were hypothesised to affect selection based on directed acyclic graph theory (appendix p 16), including age and sex of the child, location and season of death, verbal-autopsy-based cause of death, and maternal education, had to additionally meet four a priori criteria to be identified for adjustment: (1) statistically significantly associated with MITS consent (p<0·10); (2) missing less than 20% of data when comparing stillbirths and deaths of infants and children with and without MITS consent; (3) statistically significantly associated with neural tube defect as the cause of death (p<0·10); and (4) missing less than 20% of data when comparing neural tube defect and non-neural tube defect deaths. These four criteria were required only because the available aggregated HDSS data were not stratified in all possible combinations, thus necessitating a method for factor selection based on statistical significance testing and percentage missing. Factors were selected for adjustment if one or at most two factors (where age at death must be one of the two, due to data limitations) met all four criteria. If three or more factors met all four criteria, the top two were selected based on the following hierarchy: age at death, season of death, location of death, verbal autopsy cause of death, sex at birth, and maternal education. Only the child's age at death met all four criteria for adjustment (data not shown). The target population for most sites was all eligible deaths in the combined catchment areas for each respective site. However, due to the unavailability of HDSS data, the target population for Sierra Leone was all CHAMPS participants regardless of MITS consent. For Mozambique and Bangladesh, with two catchment areas each, there were two target populations: one for the catchment area where HDSS data were available and for the other, all CHAMPS participants in both catchment areas regardless of MITS consent when HDSS data were not available.

CSMFs for neural tube defects were calculated for each site as the proportion of all stillbirths and deaths of infants and children with MITS consent reviewed by the determination of cause of death panels where neural tube defect was identified within the causal chain. Adjusted CSMFs for neural tube defects, controlling for child's age, were calculated for each site using direct standardisation, where the target population was all ascertained deaths in the catchment area if HDSS data were available, or all CHAMPS deaths regardless of consent for post-mortem sampling if HDSS data were unavailable. If HDSS data in 2021 were not available, 2020 data were substituted.

If HDSS data were available, all-cause total stillbirths and mortality in children younger than 5 years (hereafter referred to as under-5 mortality) for each site was calculated as the number of stillbirths and under-5 deaths among all livebirths and stillbirths; if HDSS data were unavailable, all-cause under-5 mortality from Demographic and Health Surveys was substituted. For each site, crude and adjusted CSMFs were applied to the all-cause rate of total stillbirths and under-5 mortality to estimate the crude and adjusted cause-specific rate of total stillbirths and under-5 mortality due to neural tube defects.

All the data required to calculate the crude and adjusted CSMFs and rates of total stillbirths and under-5 mortality for each site, stratified by catchment area and age of the child, are summarised in the appendix (pp 4–11). These data included (1) numbers of deaths not enrolled in CHAMPS but that were eligible, and (2) numbers of deaths enrolled in CHAMPS who had consent for MITS, were assigned a cause of death, and who had died with a neural tube defect. Due to sparse data and better coverage properties, 90% Bayesian credible intervals (CrLs) based on weakly informative prior distributions were estimated for all crude and adjusted CSMFs and rates of total stillbirths and under-5 mortality.^23^

### Role of the funding source

The funder of the study had no role in study design, data collection, data analysis, data interpretation, or writing of the report.

## Results

**Figure 1:**
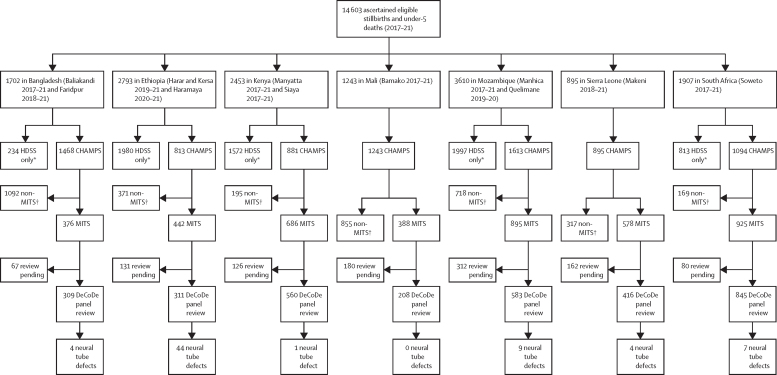
Flow diagram Flow of participants from ascertainment of stillbirth and deaths in children younger than 5 years to CHAMPS enrolment, MITS consent, cause of death determination, and neural tube defect anywhere in the causal chain, by site. Where 2021 HDSS data were not available, 2020 HDSS data were substituted. CHAMPS=Child Health and Mortality Prevention Surveillance Network. DeCoDe=determination of cause of death. MITS=minimally invasive tissue sampling. HDSS=health and demographic surveillance systems. *Eligible stillbirths and deaths in children younger than 5 years never enrolled in CHAMPS. †Deaths enrolled in CHAMPS not eligible for or refused MITS.

From Jan 1, 2017, to Dec 31, 2021, 3814 stillbirths and 10 789 deaths among children younger than 5 years were identified in the catchment areas from all seven sites. Of these, 8007 were enrolled in CHAMPS, with different enrolment rates across sites (figure 1); 2810 (35%) were stillbirths, and 5197 (65%) were children younger than 5 years. Of those enrolled, 4773 (60%) were deaths eligible for MITS whose families were approached, 4369 (92%) of whom consented to MITS, with variation across sites (Bangladesh 376 [70%] of 537, Ethiopia 442 [84%] of 526, Kenya 686 [96%] of 715, Mali 388 [97%] of 400, Mozambique 895 [95%] of 942, Sierra Leone 578 [94%] of 615, and South Africa 925 [97%] of 954). Of those who consented, MITS was done in 4290 (98%), of which 3232 (75%) were reviewed by the site's determination of cause of death panels by May 24, 2022.

Among the 3232 deaths with cause of death information, 1268 (39%) were neonates, 1119 (35%) were stillbirths, and 845 (26%) were children aged 1–59 months (table 1). Of those 3232 deaths, 296 (9%) had one or more birth defects contributing to death, and 69 (2·1% [95% CI 1·7–2·6]) were determined to have neural tube defect anywhere in the causal chain (appendix p 17).

**Table 1:** Characteristics of CHAMPS deaths consented for minimally invasive tissue sampling, determination of cause of death, and with neural tube defects anywhere in the mortality causal chain (2017–21)

		**Reviewed by determination of cause of death panel (n=3232)**	**Neural tube defects (n=69)**	p value*
**Characteristics of stillbirths, infants, and children**				
Site		..	..	<0·0001
	Bangladesh	309 (10%)	4 (6%)	..
	Ethiopia	311 (10%)	44 (64%)	..
	Kenya	560 (17%)	1 (1%)	..
	Mali	208 (6%)	0	..
	Mozambique	583 (18%)	9 (13%)	..
	Sierra Leone	416 (13%)	4 (6%)	..
	South Africa	845 (26%)	7 (10%)	..
Age group		..	..	<0·0001
	Stillbirths	1119 (35%)	51 (74%)	..
	Neonates	1268 (39%)	16 (23%)	..
	Infants and children	845 (26%)	2 (3%)	..
Sex†		..	..	<0·0001
	Female	1448 (45%)	54 (78%)	..
	Male	1780 (55%)	15 (22%)	..
Location of death†		..	..	0·0077
	Facility	2894 (90%)	68 (99%)	..
	Other	336 (10%)	1 (1%)	..
Year of death†		..	..	<0·0001
	2017	441 (14%)	3 (4%)	..
	2018	482 (15%)	2 (3%)	..
	2019	924 (29%)	16 (23%)	..
	2020	697 (22%)	19 (28%)	..
	2021	678 (21%)	29 (42%)	..
Season of death†		..	..	0·012
	Rainy	1239 (39%)	16 (23%)	..
	Dry	1983 (62%)	53 (77%)	..
Verbal autopsy cause of death†‡		..	..	0·0053
	Infection	499 (18%)	0	..
	Trauma	30 (1%)	0	..
	Other	2182 (81%)	64 (100%)	..
**Maternal characteristics**§				
Age group†		..	..	0·77
	<20 years	300 (18%)	6 (13%)	..
	20–24 years	493 (29%)	14 (29%)	..
	25–29 years	416 (25%)	14 (29%)	..
	≥30 years	467 (28%)	14 (29%)	..
Affiliated religion†		..	..	0·0022
	Christian	1459 (54%)	17 (29%)	..
	Hindu	36 (1%)	1 (2%)	..
	Muslim	1101 (41%)	41 (70%)	..
	Other	108 (4%)	0	..
Education†		..	..	0·49
	None	133 (16%)	2 (29%)	..
	Primary	308 (38%)	1 (14%)	..
	Secondary	248 (31%)	2 (29%)	..
	Tertiary	121 (15%)	2 (29%)	..
Alcohol during pregnancy†		..	..	1·0
	No	684 (99%)	16 (100%)	..
	Yes	7 (1%)	0	..
Smoking during pregnancy†		..	..	1·0
	No	696 (99%)	16 (100%)	..
	Yes	7 (1%)	0	..
Number of antenatal care visits†		..	..	0·0069
	0	128 (9%)	10 (31%)	..
	1–2	370 (27%)	7 (22%)	..
	3–4	551 (40%)	9 (28%)	..
	5–6	263 (19%)	4 (13%)	..
	7–8	47 (3%)	1 (3%)	..
	9–10	23 (2%)	1 (3%)	..

Percentages (column distributions) might not sum to 100% due to rounding. CHAMPS=Child Health and Mortality Prevention Surveillance Network. ICD-10=International Classification of Diseases, Revision 10.

* Fisher's exact test: statistically significant α=0·05.

† Missing data: determination of cause of death review: sex (n=4), location (n=2), year (n=10), season (n=10), verbal autopsy cause of death (n=521), age group (n=1556), affiliated religion (n=528), education (n=2422), alcohol during pregnancy (n=1292), smoking during pregnancy (n=1294), number of antenatal care visits (n=1850); neural tube defects: verbal autopsy cause of death (n=5), age group (n=21), affiliated religion (n=10), education (n=62), alcohol during pregnancy (n=13), smoking during pregnancy (n=13), number of antenatal clinic visits (n=37).

‡ Inter-verbal autopsy algorithm: infection (ICD-10 codes 01, 10.3–10.5); trauma (ICD-10 code 12).

§ All characteristics pertinent to time of pregnancy.

The proportion of deaths due to birth defects attributable to neural tube defects varied by site; of 208 cases reviewed by the determination of cause of death panel in Mali, 34 (16%) died due to a birth defect, but none with a neural tube defect. By contrast, of 311 cases reviewed by the determination of cause of death panel in Ethiopia, 57 (18%) died due to a birth defect, of whom 44 (77%) died with a neural tube defect. Of the 69 deaths attributable to neural tube defects, 44 (64%) occurred in Ethiopia, nine (13%) occurred in Mozambique, and seven (10%) occurred in South Africa (table 1). Among the deaths attributable to neural tube defects, 23 (33%) individuals had anencephaly, 22 (32%) had spina bifida, 19 (28%) had craniorachischisis, four (6%) had iniencephaly, and one (1%) had encephalocele (table 2). 49 (71%) deaths attributable to neural tube defects had no other comorbid causes of death (appendix 11). The comorbid causes of death among remaining deaths with a neural tube defect were perinatal asphyxia or hypoxia (14 [44%]), followed by sepsis (seven [22%]), other birth defects (three [9%]), lower respiratory tract infections (three [9%]), meningitis or encephalitis (three [9%]), and congenital infections (two [6%]).

**Table 2:** Frequency of neural tube defect subtype diagnoses among CHAMPS deaths consented for minimally invasive tissue sampling and with neural tube defects anywhere in the mortality causal chain (2017–21)

	**Bangladesh**	**Ethiopia**	**Kenya**	**Mali**	**Mozambique**	**Sierra Leone**	**South Africa**	**Total**
Anencephaly	3 (13%)	6 (26%)	1 (4)	0	7 (30%)	2 (9%)	4 (17%)	23
Craniorachischisis	0	19 (100%)	0	0	0	0	0	19
Iniencephaly	0	4 (100%)	0	0	0	0	0	4
Encephalocele	0	0	0	0	1 (100%)	0	0	1
Spina bifida	1 (5%)	15 (68%)	0	0	1 (5%)	2 (9%)	3 (14%)	22
Total	4 (6%)	44 (64%)	1 (1%)	0	9 (13%)	4 (6%)	7 (10%)	69

Includes immediate, underlying, and comorbid causes of death. CHAMPS=Child Health and Mortality Prevention Surveillance Network.

**Figure 2:**
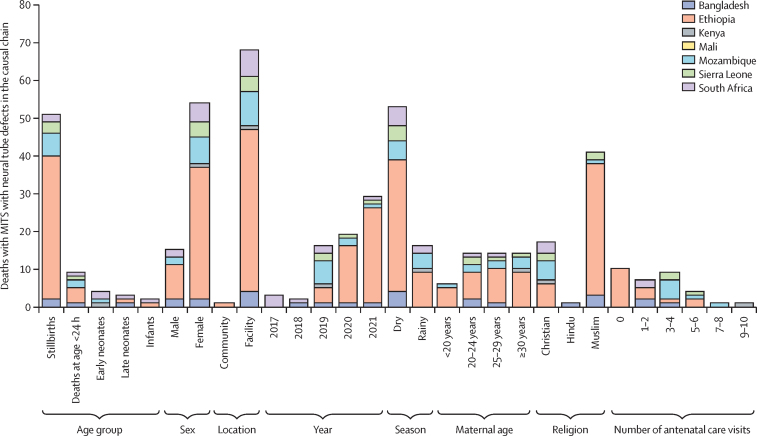
Characteristics of CHAMPS deaths consented for MITS and with fatal neural tube defects anywhere in the mortality causal chain, by site (n=69) Missing data for maternal age: Bangladesh n=1; Ethiopia n=14; Mozambique n=1; South Africa n=5. Missing data for religion: Ethiopia n=3; Mozambique n=3; South Africa n=4. Missing data for number of antenatal care visits: Bangladesh n=1; Ethiopia n=28; Mozambique n=2; Sierra Leone n=1; South Africa n=5. CHAMPS=Child Health and Mortality Prevention Surveillance Network. MITS=minimally invasive tissue sampling.

Among all deaths enrolled in CHAMPS, a significantly higher proportion with a neural tube defect were enrolled in Ethiopia than in any other country; were stillbirths compared with other age groups; were male and not female; occurred in a health facility not in the community; occurred in 2021 than any other year; occurred during the dry season compared with the rainy season; and had a verbal autopsy-based cause of death other than infection or trauma (table 1; figure 2; appendix 12). The mothers of individuals who died with a neural tube defect were more likely to be Muslim than of any other religion, and were less likely to attend antenatal care (table 1; appendix p 12). A multivariable logistic regression analysis to identify risk factors for death with a neural tube defect showed that being enrolled in Ethiopia (adjusted OR 8·09 [95% CI 2·84–23·0]), being female (4·40 [2·44–7·93]), and having a mother who did not attend antenatal care (2·48 [1·12–5·51]), were more commonly observed among deaths with a neural tube defect in the causal chain (table 3).

**Table 3:** Multivariable logistic regression analysis to identify risk factors for death with a neural tube defect in the causal chain among stillbirths and children younger than 5 years in the CHAMPS Network (2017–21)

		Adjusted OR*†**(95% CI)**	**p value**
**Characteristics of stillbirths, infants, and children**			
Site‡			
	Bangladesh	0·32 (0·06–1·80)	0·19
	Ethiopia	8·09 (2·84–23·02)	<0·0001
	Kenya	0·17 (0·02–1·50)	0·11
	Mali§	0·10 (0·00–0·50)	0·0063
	Mozambique	0·78 (0·27–2·23)	0·65
	Sierra Leone	0·61 (0·14–2·58)	0·50
	South Africa	1 (ref)	..
Sex¶			
	Male	1 (ref)	..
	Female	4·40 (2·44–7·93)	<0·0001
**Maternal characteristics**‖			
Age group**			
	<20 years	2·10 (0·80–5·54)	0·13
	20–24 years	1·99 (0·96–4·09)	0·063
	25–29 years	1·80 (0·89–3·64)	0·10
	≥30 years	1 (ref)	..
Religion††			
	Christian	1 (ref)	..
	Hindu	1·85 (0·17–20·02)	0·61
	Muslim	0·62 (0·29–1·31)	0·21
	Other§	0·26 (0·00–1·19)	0·079
Education‡‡			
	None	0·83 (0·19–3·67)	0·80
	Primary	0·38 (0·04–3·44)	0·39
	Secondary	0·67 (0·13–3·44)	0·64
	Tertiary	1 (ref)	..
Alcohol§			
	Yes	4·50 (0·00–22·26)	0·85
	No	1 (ref)	..
Smoking§			
	Yes	5·61 (0·00–28·20)	0·88
	No	1 (ref)	..
Number of antenatal care visits§§			
	0	2·48 (1·12–5·51)	0·026
	1–2	1·37 (0·56–3·31)	0·49
	3–4	1·11 (0·50–2·47)	0·79
	5–6	1·41 (0·46–4·29)	0·54
	7–8	2·25 (0·28–17·98)	0·45
	9–10	1 (ref)	..

CHAMPS=Child Health and Mortality Prevention Surveillance Network. OR=odds ratio.

* Stillbirths, infants, and children who did not die due to a neural tube defect did die but due to some cause other than a neural tube defect.

† Adjusted ORs and 95% CIs from reduced models in which all possible subsets of potential confounders (site of death; age and sex of the child; age, education, and religion of the mother; and antenatal care visits) had been dropped were compared with those from the fully adjusted model; if it was within 10% of the fully adjusted model, there was evidence that the variable was not a confounder and was dropped.

‡ Adjusted for age and sex of the child; age, education, and religion of the mother; and antenatal care visits.

§ Exact logistic regression model performed due to sparse data.

¶ Adjusted for site of death; age of the child; age, education, and religion of the mother; and antenatal care visits.

‖ All characteristics pertinent to time of pregnancy.

** Adjusted for site of death; age and sex of the child; education and religion of the mother; and antenatal care visits.

†† Adjusted for site of death; age and sex of the child; age and education of the mother; and antenatal care visits.

‡‡ Adjusted for site of death; age and sex of the child; age and religion of the mother; and antenatal care visits.

§§ Adjusted for site of death; age and sex of the child; and age, education, and religion of the mother.

**Figure 3:**
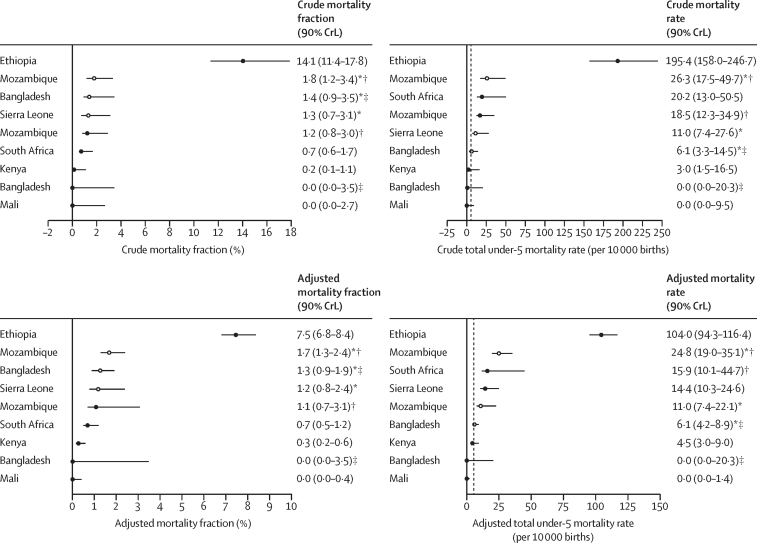
Crude and adjusted cause-specific mortality fractions and total under-5 mortality due to fatal neural tube defects among all stillbirths and deaths in children younger than 5 years in the target population by site, controlling for age Dashed line denotes expected rate of 5–6 deaths per 10 000 births when an effective national folic acid fortification programme is implemented. CHAMPS=Child Health and Mortality Prevention Surveillance Network. CrL=Bayesian credible interval. HDSS=health and demographic surveillance systems. *Target population consists of all deaths enrolled in CHAMPS, regardless of minimally invasive tissue sampling consent. †Quelimane was included in the estimate where the target population was all CHAMPS deaths but excluded when it was the catchment area because HDSS data were not available. ‡Faridpur was included in the estimate where the target population was all CHAMPS deaths but excluded when it was the catchment area because HDSS data were not available.

The age-adjusted mortality fraction of CHAMPS deaths attributed to neural tube defects ranged from 0·0% (90% CrL 0·0–0·4) in Mali to 7·5% (6·8–8·4) in Ethiopia. For all estimates, crude and adjusted, Ethiopia had the highest mortality fraction of deaths due to neural tube defects among all the sites, 4–25 times greater than any other sites. Similarly, after adjustment by age, an estimated 104·0 per 10 000 births (94·3–116·4) in Ethiopia died due to a neural tube defect, 4–23 times greater than any other sites (figure 3). Bangladesh and Mali were estimated to have a negligible mortality rate attributed to neural tube defects.

## Discussion

This real-time mortality surveillance of stillbirths and children younger than 5 years conducted in seven sites in sub-Saharan Africa and southeast Asia found an overall proportion of neural tube defects of 2·1% among 3232 deaths reviewed across CHAMPS sites. Ethiopia had the highest mortality fraction, 7·5% (95% CI 6·8–8·4), and mortality attributed to neural tube defects, 104·0 per 10 000 births (95% CI 94·3–116·4). Furthermore, our study found that deaths from neural tube defects were more likely to be in female individuals, have mothers who did not attend antenatal care, and be in Ethiopia.

Some geographical heterogeneity in deaths with neural tube defects found in this study might be explicable by methodological differences and potential biases, such as the differential number of deaths who were enrolled and consented to MITS among babies with neural tube defects by site. However, our estimates on all-cause total stillbirths and under-5 mortality in South Africa, Mozambique, and Sierra Leone were within the range of the estimates of stillbirths and child mortality (15·3 per 10 000 births [95% CI 10·2–20·3]) among individuals with neural tube defects in sub-Saharan African countries with fortification with folic acid as reported by Blencowe and colleagues.^12^ By contrast, our estimates suggest that Kenya and Mali had a lower mortality attributed to neural tube defects than the average for the region for countries with mandatory programmes of fortified wheat or maize with folic acid.^12^ The coverage of mandatory fortification of food vehicles is not well reported in these countries,^24^ and there are no clear data on the effect on the prevalence of neural tube defects after implementing mandatory food fortification programmes with folic acid. However, it is known that in Kenya and Mali, 90–100% of industrially milled flour is fortified with folic acid,^24^ and a small survey in Kenya suggested that pregnant women had a high awareness of fortified foods along with a high (80%) study participant-reported coverage of consumption of fortified food with folic acid,^25^ which might explain the low total stillbirths and under-5 mortality noted in our study. In Bangladesh, when considering the CHAMPS population, we found a rate of total stillbirths and under-5 mortality due to neural tube defects of 6·1 per 10 000 births, similar to the previously reported modelled estimate in southeast Asia.^12^ When we excluded Faridpur in Bangladesh due to an absence of HDSS data, the attributable mortality to neural tube defects was negligible, which could be explained by a geographical difference in risk and severity of neural tube defects, or by the higher burden of neural tube defects in the Faridpur CHAMPS site, which has a tertiary care hospital and might therefore be more likely to be referred complicated cases than the other CHAMPS catchment in Bangladesh. The mortality attributable to neural tube defects of 104·0 per 10 000 births found in Ethiopia is eight times higher than the average in sub-Saharan Africa.^12^ However, the adjusted total stillbirths and under-5 mortality attributed to neural tube defects in the catchment area is probably underestimated because all the deaths due to neural tube defects found in Ethiopia by CHAMPS were facility deaths; stillbirths, neonatal deaths, and babies with neural tube defects who died at home or those children dying outside of the neonatal period are not well represented.

Despite the high prevalence of neural tube defects already reported for Ethiopia,^14^, ^26^ there is no similar literature on contribution of neural tube defects to all-cause mortality that could be used for comparison.^27^ We found that neural tube defects occurred more frequently among female individuals, similar to reports from previous studies. We also found that most neural tube defects in Ethiopia were craniorachischisis, whereas no craniorachischisis was found in other sites. There is no previous literature reporting a similar finding. However, several studies have found a higher prevalence in Ethiopia of severe neural tube defects, such as anencephaly, whereas in many other countries, spina bifida is more prevalent.^28^, ^29^ Income level, diet, and folic acid fortification are suggested as the main factors related to the prevalence of severe neural tube defects in any one country and might explain regional variation.^28^, ^29^ However, both mortality estimates and factors associated with mortality found in the current study cannot be easily compared with previous studies because CHAMPS has a unique methodology of mortality surveillance, studying only neural tube defects with fatal outcomes and using post-mortem sampling and clinical review for careful diagnosis of causes of death.

Estimates suggest that folic acid food fortification might reduce the prevalence of neural tube defects by 46% (95% CI 37–54) and prevent 13% of all neonatal deaths worldwide.^30^ According to the Global Fortification Data Exchange, all countries where CHAMPS is working have either mandatory or voluntary food fortification programmes with varying levels of folic acid added to wheat flour, maize flour, or rice, and with varying programme performance ranging from 0% to 90% (appendix p 15).^8^ Several countries where CHAMPS is operating (ie, Bangladesh, Ethiopia, and Sierra Leone) did not have mandatory food fortification policies with folic acid during the study period (appendix p 15). It has been established that voluntary food fortification does not yield an effective prevention return compared with mandatory food fortification.^11^ Our results suggest that countries where CHAMPS is working with mandatory folic acid fortification, including Kenya and Mali, have high fortification programme coverage, and are averting most cases of folic acid-preventable neural tube defects, whereas South Africa and Mozambique can improve their fortification programmes to achieve better prevention of neural tube defects.^31^

Food fortification is highly cost saving for low-income and lower-middle-income countries. A cost-effectiveness analysis after implementation of food fortification with folic acid showed the intervention cost per individual with a neural tube defect was 1200 international dollars and the intervention cost per infant death averted was 11 000 international dollars, and these averted costs of care yielded a net saving of 2·3 million international dollars.^32^ Similarly, in South Africa, one of the CHAMPS sites, the cost–benefit ratio of fortification in terms of averted neural tube defects after fortification was 1:46.^16^ A 2018 economic analysis presented compelling data supporting investment in fortification, especially for governments with limited resources and multiple competing priorities.^33^ In this analysis, the estimated cost per death averted through mandatory fortification was US$957 and the cost per averted disability-adjusted life year was $15. Both estimates were similar to other life-saving public health strategies such as rotavirus vaccines and insecticide-treated bed nets for Malaria prevention.^33^

Our analysis has some limitations. First, most of the CHAMPS deaths with cause of death information and all neural tube defects occurred in a health facility. We adjusted the data to estimate mortality in the catchment area using HDSS denominators that included all deaths, but mortality due to neural tube defects might still be underestimated due to the absence of neural tube defects recorded at the community level. Some families might have been unwilling to report the death to the CHAMPS team when the cause of death was clearly a birth defect. Second, the majority of the neural tube defects detected by CHAMPS were stillbirths and neonatal deaths. It is known that all anencephaly results in stillbirth or death in the first days of life; 25% of individuals with spina bifida will die during the neonatal period. Among individuals with spina bifida who survive the neonatal period, 75% do not survive beyond the age of 5 years.^31^ With this understanding, we would expect to find more deaths related to neural tube defects, specifically spina bifida, among infants and older children. Difficulties in obtaining consent to enrol older children who died, especially at the community level, are likely to have caused our findings to underestimate the true incidence in this population. Third, we found marked geographical heterogeneity in the proportion of deaths that were caused by neural tube defects that could be also explained by variable enrolment of deceased children and consent for MITS across CHAMPS sites. Neural tube defects are visible defects and, in some countries, might lead parents hide the death or, on the contrary, be more willing to consent to understand the cause of the birth defect. This factor could produce very different mortality with neural tube defects by site. We could not assess consent rate for MITS among birth defects across sites because no information for non-consented cases was collected. Fourth, we did not explore the different causes of neural tube defects in this study. However, the high proportion of birth defects found in CHAMPS has led the network to include a pilot genomic assessment in specific cases as part of the study procedures. Fifth, although the use of statistical significance testing and percentage missing criteria to further select the factors for adjustment were necessary due to how the available HDSS data were structured, this might exclude important factors that contribute to selection bias. Sixth, estimates might be susceptible to sparse data bias and unmeasured confounding, as the relatively small number of neural tube defects was made smaller after stratification, and only factors that had been measured both in CHAMPS and HDSS were eligible for adjustment. Finally, the adjustment to estimate mortality fractions and total stillbirths and under-5 mortality for each catchment area was not standardised across sites given the differences among HDSS data. However, we used the most reliable mortality data available at all sites, and the majority were HDSS data.

Our findings highlight neural tube defects as a relevant cause of death in stillbirths, infants, and children younger than 5 years, emphasising the importance of implementing or improving mandatory national food fortification programmes with folic acid to reduce the prevalence of neural tube defects and associated mortality. The findings from Ethiopia, in particular, are alarming, and urgent implementation of food fortification with folic acid could reduce the prevalence of neural tube defects. Continued monitoring of mandatory fortification programmes that are already functional in countries where CHAMPS is active could identify and address coverage and fortification quality limitations to effectively prevent all folate-sensitive neural tube defects.

## Data sharing

Summarised data are publicly available through the CHAMPS website: https://champshealth.org/data/enrolled-population-summary/. Requests for further detailed data for research and evaluation purposes can be made at: https://champshealth.org/data/.

## Declaration of interests

SEA reports grants from Emory University, during the conduct of the study. JAGS reports grants from the Wellcome Trust, the National Institute for Health and Care Research, Gavi, the Foreign Commonwealth and Development Office (UK), the European and Developing Countries Clinical Trials Partnership, the Medical Research Council, and the Bill & Melinda Gates Foundation whose payments were direct to his institution. LM reports grants from the Bill & Melinda Gates Foundation via Emory, whose payments were made direct to her institution. KLK reports grants from the Bill & Melinda Gates Foundation, whose payments were made direct to her institution and support attending meetings. KLK, SOS, and AMK report the provision of study materials and funding to their institution for this study from the Bill & Melinda Gates Foundation. AW reports a stipend for participating in the CHAMPS research and payment for travel to the High Horizons meeting in Belgium from the University of Witwatersrand; honoraria for lectures from Sanofi; and being a council member of The South African Society of Obstetricians and Gynaecologists. All other authors declare no competing interests.
