# Supplementary material for: Neural tube defects as a cause of death among stillbirths, infants, and children younger than 5 years in sub-Saharan Africa and southeast Asia: an analysis of the CHAMPS network
Source: Lancet Glob Health. 2023 Jun 1;11(7):e1041–52. doi: 10.1016/S2214-109X(23)00191-2 (PMC10282076; doi:10.1016/S2214-109X(23)00191-2)
Supplement: Supplementary appendix [file mmc1.pdf]

THE LANCET
Global Health

Supplementary appendix

This appendix formed part of the original submission and has been peer reviewed. We post it as supplied by the authors.

Supplement to: Madrid L, Vyas KJ, Kancherla V, et al. Neural tube defects as a cause of death among stillbirths, infants, and children younger than 5 years in sub-Saharan Africa and southeast Asia: an analysis of the CHAMPS network. *Lancet Glob Health* 2023; published online June 1. https://doi.org/10.1016/S2214-109X(23)00191-2.

Appendix

Neural tube defects as a cause of death among stillbirths and children under five in Sub-Saharan Africa and South-East Asia – A population-based analysis of the CHAMPS network.

Supplemental Table 1. Published studies on the prevalence of neural tube defects in CHAMPS sites.

		Surveillance characteristics			Pregnancy outcomes surveyed		Prevalence (per 10 000 births)		
Site	Author	Region	Period	Source	Numerator	Denominator	All NTDs	Anencephaly	Spina bifida
Bangladesh	Dey et al., 2010	Dhaka	2006-2007	Hospital	LB	LB	15	3	0
Ethiopia	Kindie et al., 2022	North Shewa	2017-2019	Hospital	LB, ETOPFA, SB	LB, ETOPFA, SB	109	56	35
	Berihu et al., 2018	Tigray	2016-2017	Hospital	LB, SB	LB, SB	131	66	64
	Seyoum et al., 2018	Amhara	2015-2017	Hospital	LB, SB	LB, SB	36	5	31
	Gedefaw et al., 2018	Addis Ababa	2016-2016	Hospital	LB, SB	LB, SB	128	69	52
	Sorri et al., 2015	Addis Ababa	2009-2012	Hospital	LB, SB	LB, SB	61	27	33
	Taye et al., 2019	Addis Ababa, Amhara	2015-2015	Hospital	LB	LB	48	5	43
	Taye et al., 2016	Addis Ababa, Amhara	2010-2014	Hospital	LB	LB	38	6	31
	Mekonen et al., 2015	Tigray	2011-2012	Hospital	LB	LB	132	7	125
	Abdu et al., 2019	Amhara	2015-2017	Hospital	LB	LB	59		53
	Abebe et al., 2019	Oromia	2011-2015	Hospital	LB, SB	LB, SB	26	14	11
	Mitiku et al., 2017	Addis Ababa	2017-2017	Hospital	LB, ETOPFA, SB	LB, ETOPFA, SB	281	238	
	Adane et al., 2018	Amhara	2015-2017	Hospital	LB, SB	LB, SB	52	4	27
	Legesse et al., 2019	Addis Ababa	2018-2019	Hospital	LB, SB	LB, SB	63		
Kenya	Githuku et al., 2014	Kijabe	2005-2010	Hospital	LB, SB	LB	3		
	Muga et al., 2009	Nairobi	1983-1984	Hospital	LB, SB	LB, SB	50		
Mali[1]									
Mozambique[1]									
Sierra Leone[1]									
South Africa	Sayed et al., 2008	Eastern Cape, KwaZulu Natal, Mpumalanga, Free State	2004-2005	Hospital	LB, SB	LB, SB	10	4	5
	Krzesinski et al., 2019	Western Cape	2003-2013	Hospital	LB, SB	LB, SB	8	3	3

ETOPFA, elective termination of pregnancy for fetal anomalies; LB, live-births; NTD, neural tube defects; SB, stillbirths.
[1]Mali, Mozambique, and Sierra Leone did not have any published studies on NTD prevalence.

DEFINITIONS.

- **Stillbirths:** no spontaneous breathing or movement at the time of delivery and [1] weighing >1 kg or [2] estimated gestational age ≥28 weeks
- **Neonates:** live-born babies aged 1–28 days
- **Infants:** aged 29–364 days
- **Children:** aged 1–5 years
- **Seasonality:**
    - The Bangladesh dry season was defined as November– May and the rainy season as June–October;
    - the Ethiopia dry season was October–May and the rainy season was June–September;
    - the Kenya dry season was July and December–March and the rainy season was April–June and August–November;
    - the Mali dry season was November–May and the rainy season was June–October;
    - the Mozambique dry season was May–October and the rainy season was November–April;
    - the Sierra Leone dry season was October–May and the rainy season was June– September;
    - the South Africa dry season was March– November and the rainy season was December–February

BANGLADESH

Supplemental Table 2. Frequency of deaths in the target population that are enrolled in CHAMPS, consented for MITS and are DeCoDed as of May 24, 2022, and attributed neural tube defect as a cause of death anywhere in the causal chain, by study period and age.

Site	Age	CHAMPS	Target	MITS	NTD	Catchment	Age	DSS only	CHAMPS	Target	MITS	NTD	Live-births	Catchment	Age	CHAMPS	Target	MITS	NTD
Bangladesh	2017					Baliakandi	2017							Faridpur	2017				
	Stillbirth	28	28	0	0		Stillbirth	1	28	29	0	0			Stillbirth				
	Neonate	38	38	5	0		Neonate	1	38	39	5	0	4787[1]		Neonate				
	Infant/Child	10	10	0	0		Infant/Child	0	10	10	0	0			Infant/Child				
	2018						2018								2018				
	Stillbirth	136	136	20	0		Stillbirth	18	126	144	10	0			Stillbirth	10	10	10	0
	Neonate	113	113	19	1		Neonate	7	105	112	11	0	4078		Neonate	8	8	8	1
	Infant/Child	44	44	1	0		Infant/Child	4	44	48	1	0			Infant/Child	0	0	0	0
	2019						2019								2019				
	Stillbirth	159	159	53	0		Stillbirth	21	117	138	11	0			Stillbirth	42	42	42	0
	Neonate	178	178	55	1		Neonate	28	148	176	25	0	5193		Neonate	30	30	30	1
	Infant/Child	46	46	2	0		Infant/Child	6	44	50	0	0			Infant/Child	2	2	2	0
	2020						2020								2020				
	Stillbirth	177	177	26	1		Stillbirth	47	161	208	10	0			Stillbirth	16	16	16	1
	Neonate	152	152	30	0		Neonate	19	132	151	10	0	5090		Neonate	20	20	20	0
	Infant/Child	47	47	0	0		Infant/Child	8	47	55	0	0			Infant/Child	0	0	0	0
	2021						2021[2]								2021				
	Stillbirth	149	149	49	1		Stillbirth	47	85	132	12	0			Stillbirth	64	64	37	1
	Neonate	146	146	48	0		Neonate	19	80	99	6	0	5090[2]		Neonate	66	66	42	0
	Infant/Child	43	43	1	0		Infant/Child	8	42	50	0	0			Infant/Child	1	1	1	0
	2017-2021						2017-2021								2018-2021				
	Stillbirth	649	649	148	2		Stillbirth	134	517	651	43	0			Stillbirth	132	132	105	2
	Neonate	627	627	157	2		Neonate	74	503	577	57	0	24 238		Neonate	124	124	100	2
	Infant/Child	190	190	4	0		Infant/Child	26	187	213	1	0			Infant/Child	3	3	3	0

[1]Imputed simple average using all the years for which live-birth data were available.

[2]2021 DSS data not available; substituted 2020 DSS data.

ETHIOPIA

Supplemental Table 3. Frequency of deaths in the target population that are enrolled in CHAMPS, consented for MITS and are DeCoDed as of May 24, 2022, and attributed neural tube defect as a cause of death anywhere in the causal chain, by study period and age.

Site	Age	DSS only	CHAMPS	Target	MITS	NTD	Live-births
Ethiopia	2019						
	Stillbirth	75	33	108	22	1	
	Neonate	62	37	99	17	2	3022
	Infant/Child	477	48	525	12	1	
	2020						
	Stillbirth	85	93	178	74	14	
	Neonate	58	67	125	39	1	7995
	Infant/Child	540	44	584	8	0	
	2021[1]						
	Stillbirth	85	217	302	103	23	
	Neonate	58	129	187	32	2	7995[1]
	Infant/Child	540	71	611	5	0	
	2019-2021						
	Stillbirth	245	343	588	199	38	
	Neonate	178	233	411	88	5	19 012
	Infant/Child	1557	163	1720	25	1	

Catchment	Age	DSS only	CHAMPS	Target	MITS	NTD	Live-births
Haramaya	2019						
	Stillbirth						
	Neonate						
	Infant/Child						
	2020						
	Stillbirth	32	32	64	27	9	
	Neonate	4	14	18	8	0	3210
	Infant/Child	158	6	164	1	0	
	2021[1]						
	Stillbirth	32	74	106	43	14	
	Neonate	4	26	30	11	1	3210[1]
	Infant/Child	158	3	161	1	0	
	2019-2021						
	Stillbirth	64	106	170	70	23	
	Neonate	8	40	48	19	1	6420
	Infant/Child	316	9	325	2	0	

Catchment	Age	DSS only	CHAMPS	Target	MITS	NTD	Live-births
Harar	2019						
	Stillbirth	8	19	27	15	1	
	Neonate	1	17	18	13	2	469
	Infant/Child	10	3	13	3	0	
	2020						
	Stillbirth	4	34	38	27	1	
	Neonate	1	31	32	24	1	768
	Infant/Child	12	0	12	0	0	
	2021[1]						
	Stillbirth	4	34	38	17	1	
	Neonate	1	21	22	8	1	768[1]
	Infant/Child	12	1	13	0	0	
	2019-2021						
	Stillbirth	16	87	103	59	3	
	Neonate	3	69	72	45	4	2005
	Infant/Child	34	4	38	3	0	

Catchment	Age	DSS only	CHAMPS	Target	MITS	NTD	Live-births
Kersa	2019						
	Stillbirth	67	14	81	7	0	
	Neonate	61	18	79	4	0	2553
	Infant/Child	467	44	511	9	1	
	2020						
	Stillbirth	49	27	76	20	4	
	Neonate	53	22	75	7	0	4017
	Infant/Child	370	38	408	7	0	
	2021[1]						
	Stillbirth	49	109	158	43	8	
	Neonate	53	82	135	13	0	4017[1]
	Infant/Child	370	67	437	4	0	
	2019-2021						
	Stillbirth	165	150	315	70	12	
	Neonate	167	122	289	24	0	10 587
	Infant/Child	1207	149	1356	20	1	

[1]2021 DSS data not available; substituted 2020 DSS data.

KENYA

Supplemental Table 4. Frequency of deaths in the target population that are enrolled in CHAMPS, consented for MITS and are DeCoDed as of May 24, 2022, and attributed neural tube defect as a cause of death anywhere in the causal chain, by study period and age.

Site	Age	DSS only	CHAMPS	Target	MITS	NTD	Live-births
Kenya	2017						
	Stillbirth	52	21	73	11	0	
	Neonate	102	16	118	8	0	3129
	Infant/Child	429	43	472	29	0	
	2018						
	Stillbirth	14	51	65	29	0	
	Neonate	64	77	141	43	0	3283
	Infant/Child	219	101	320	62	0	
	2019						
	Stillbirth	15	65	80	52	0	
	Neonate	61	70	131	51	1	3146
	Infant/Child	256	105	361	88	0	
	2020						
	Stillbirth	27	48	75	40	0	
	Neonate	64	49	113	40	0	3186
	Infant/Child	89	37	126	28	0	
	2021[2]						
	Stillbirth	27	54	81	16	0	
	Neonate	64	58	122	25	0	3186[2]
	Infant/Child	89	84	173	24	0	
	2017-2021						
	Stillbirth	135	239	374	148	0	
	Neonate	355	270	625	167	1	15 930
	Infant/Child	1082	370	1452	231	0	

Catchment	Age	DSS only	CHAMPS	Target	MITS	NTD	Live-births
Manyatta	2017						
	Stillbirth	0	11	11	10	0	
	Neonate	2	9	11	7	0	1196[1]
	Infant/Child	4	24	28	23	0	
	2018						
	Stillbirth	1	24	25	19	0	
	Neonate	11	35	46	28	0	1233
	Infant/Child	27	36	63	32	0	
	2019						
	Stillbirth	2	25	27	23	0	
	Neonate	9	32	41	31	0	1158
	Infant/Child	26	41	67	39	0	
	2020						
	Stillbirth	1	19	20	17	0	
	Neonate	30	31	61	25	0	1196[1]
	Infant/Child	17	18	35	15	0	
	2021[2]						
	Stillbirth	1	24	25	7	0	
	Neonate	30	31	61	18	0	1196[2]
	Infant/Child	17	33	50	9	0	
	2017-2021						
	Stillbirth	5	103	108	76	0	
	Neonate	82	138	220	109	0	5979
	Infant/Child	91	152	243	118	0	

Catchment	Age	DSS only	CHAMPS	Target	MITS	NTD	Live-births
Siaya	2017						
	Stillbirth	52	10	62	1	0	
	Neonate	100	7	107	1	0	1933
	Infant/Child	425	19	444	6	0	
	2018						
	Stillbirth	13	27	40	10	0	
	Neonate	53	42	95	15	0	2050
	Infant/Child	192	65	257	30	0	
	2019						
	Stillbirth	13	40	53	29	0	
	Neonate	52	38	90	20	1	1988
	Infant/Child	230	64	294	49	0	
	2020						
	Stillbirth	26	29	55	23	0	
	Neonate	34	18	52	15	0	1990[1]
	Infant/Child	72	19	91	13	0	
	2021[2]						
	Stillbirth	26	30	56	9	0	
	Neonate	34	27	61	7	0	1990[2]
	Infant/Child	72	51	123	15	0	
		2017-2021					
	Stillbirth	130	136	266	72	0	
	Neonate	273	132	405	58	1	9951
	Infant/Child	991	218	1209	113	0	

[1]Imputed simple average using all the years for which live-birth data were available.
[2]2021 DSS data not available; substituted 2020 DSS data.

MALI

Supplemental Table 5. Frequency of deaths in the target population that are enrolled in CHAMPS, consented for MITS and are DeCoDed as of May 24, 2022, and attributed neural tube defect as a cause of death anywhere in the causal chain, by study period and age.

Catchment	Age	CHAMPS	Target[1]	MITS	NTD	Live-births
Bamako		2017				
	Stillbirth	40	40	4	0	6708
	Neonate	58	58	9	0	
	Infant/Child	61	61	12	0	
		2018				
	Stillbirth	121	121	26	0	6624
	Neonate	106	106	32	0	
	Infant/Child	94	94	12	0	
		2019				
	Stillbirth	172	172	32	0	7602
	Neonate	136	136	24	0	
	Infant/Child	144	144	19	0	
		2020				
	Stillbirth	37	37	8	0	6978[2]
	Neonate	33	33	7	0	
	Infant/Child	31	31	8	0	
	2021[3]					
	Stillbirth	79	79	11	0	6978[3]
	Neonate	91	91	3	0	
	Infant/Child	40	40	1	0	
		2017-2021				
	Stillbirth	449	449	81	0	34 890
	Neonate	424	424	75	0	
	Infant/Child	373	373	52	0	

[1]Includes DSS only data.

[2]Imputed simple average using all the years for which live-birth data were available.

[3]2021 DSS data not available; substituted 2020 DSS data.

MOZAMBIQUE

Supplemental Table 6. Frequency of deaths in the target population that are enrolled in CHAMPS, consented for MITS and are DeCoDed as of May 24, 2022, and attributed neural tube defect as a cause of death anywhere in the causal chain, by study period and age.

Site	Age	CHAMPS	Target	MITS	NTD	Catchment	Age	DSS only	CHAMPS	Target	MITS	NTD	Live-births	Catchment	Age	CHAMPS	Target	MITS	NTD
Mozambique	2017					Manhica	2017							Quelimane	2017				
	Stillbirth	38	38	20	0		Stillbirth	26	38	64	20	0			Stillbirth				
	Neonate	74	74	28	0		Neonate	99	74	173	28	0	5459		Neonate				
	Infant/Child	127	127	26	0		Infant/Child	173	127	300	26	0			Infant/Child				
	2018						2018								2018				
	Stillbirth	62	62	25	0		Stillbirth	71	62	133	25	0			Stillbirth				
	Neonate	65	65	28	0		Neonate	85	65	150	28	0	5615		Neonate				
	Infant/Child	79	79	16	0		Infant/Child	165	79	244	16	0			Infant/Child				
	2019						2019								2019				
	Stillbirth	117	117	69	3		Stillbirth	72	65	137	28	1			Stillbirth	52	52	41	2
	Neonate	145	145	86	3		Neonate	110	72	182	24	1	5567		Neonate	73	73	62	2
	Infant/Child	105	105	39	0		Infant/Child	306	78	384	14	0			Infant/Child	27	27	25	0
	2020						2020								2020				
	Stillbirth	169	169	41	1		Stillbirth	112	62	219	26	1			Stillbirth	62	62	15	0
	Neonate	132	132	17	0		Neonate	68	60	128	9	0	5248		Neonate	72	72	8	0
	Infant/Child	99	99	20	0		Infant/Child	265	69	334	11	0			Infant/Child	30	30	9	0
	2021						2021[1]								2021				
	Stillbirth	139	139	20	1		Stillbirth	112	139	251	20	1			Stillbirth				
	Neonate	170	170	7	0		Neonate	68	170	238	7	0	5248[1]		Neonate				
	Infant/Child	71	71	6	0		Infant/Child	265	71	336	6	0			Infant/Child				
	2017-2021						2017-2021								2019-2020				
	Stillbirth	525	525	175	5		Stillbirth	393	411	804	119	3			Stillbirth	114	114	56	2
	Neonate	586	586	166	3		Neonate	430	441	871	96	1	21 889		Neonate	145	145	70	2
	Infant/Child	481	481	107	0		Infant/Child	1174	424	1598	73	0			Infant/Child	57	57	34	0

[1] 2021 DSS data not available; substituted 2020 DSS data.

SIERRA LEONE

Supplemental Table 7. Frequency of deaths in the target population that are enrolled in CHAMPS, consented for MITS and are DeCoDed as of May 24, 2022, and attributed neural tube defect as a cause of death anywhere in the causal chain, by study period and age.

Catchment	Age	CHAMPS	Target	MITS	NTD
Makeni	2018				
	Stillbirth	59	59	0	0
	Neonate	35	35	0	0
	Infant/Child	52	52	0	0
	2019				
	Stillbirth	77	77	47	1
	Neonate	81	81	51	1
	Infant/Child	110	110	74	0
	2020				
	Stillbirth	41	41	22	1
	Neonate	23	23	8	0
	Infant/Child	54	54	25	0
	2021				
	Stillbirth	63	63	37	1
	Neonate	49	49	34	0
	Infant/Child	68	68	41	0
	2019-2021				
	Stillbirth	181	181	106	3
	Neonate	153	153	93	1
	Infant/Child	232	232	140	0

SOUTH AFRICA

Supplemental Table 8. Frequency of deaths in the target population that are enrolled in CHAMPS, consented for MITS and are DeCoDed as of May 24, 2022, and attributed neural tube defect as a cause of death anywhere in the causal chain, by study period and age.

Catchment	Age	DSS only	CHAMPS	Target	MITS	NTD	Live-births
Soweto	2017						
	Stillbirth	44	61	105	1	0	3000[1]
	Neonate	47	220	267	192	3	
	Infant/Child	61	102	163	98	0	
	2018						
	Stillbirth	52	47	99	42	0	2236[1]
	Neonate	62	90	152	81	0	
	Infant/Child	76	47	123	45	1	
	2019						
	Stillbirth	45	46	91	42	1	1069[1]
	Neonate	37	41	78	39	1	
	Infant/Child	37	27	64	26	0	
	2020						
	Stillbirth	37	54	91	30	0	1233[1]
	Neonate	89	78	167	45	0	
	Infant/Child	50	30	80	15	0	
	2021[2]						
	Stillbirth	37	98	135	57	1	1233[2]
	Neonate	89	94	183	56	0	
	Infant/Child	50	59	109	35	0	
	2017-2021						
	Stillbirth	215	306	521	172	2	8771
	Neonate	324	523	847	413	4	
	Infant/Child	274	265	539	219	1	

[1]Imputed for years 2017-2018; DSS data for years 2019-2020.
[2]2021 DSS data not available; substituted 2020 DSS data.

Supplemental Table 9. Crude and adjusted mortality fractions and rates due to neural tube defects by site, controlling for age.

						Cause-specific (per 10 000)	
Site	Year	MITS	NTD	cCSMF (%) (90% Bayesian CrI)	aCSMF (%) (90% Bayesian CrI)	cTU5MR (90% Bayesian CrI)	aTU5MR (90% Bayesian CrI)
Bangladesh							
Target: CHAMPS	2017-2021	309	4	1·3 (0·7, 3·1)	1·3 (0·9, 1·9)	6·1 (3·3, 14·5)	6·1 (4·2, 8·9)
Target: Catchment(s)	2017-2021	101	0	0·0 (0·0, 3·5)	0·0 (0·0, 3·5)	0·0 (0·0, 20·3)	0·0 (0·0, 20·3)
Ethiopia	2019-2021	312	44	14·1 (11·4, 17·8)	7·5 (6·8, 8·4)	195·4 (158·0, 246·7)	104·0 (94·3, 116·4)
Kenya	2017-2021	546	1	0·2 (0·1, 1·1)	0·3 (0·2, 0·6)	3·0 (1·5, 16·5)	4·5 (3·0, 9·0)
Mali	2017-2021	208	0	0·0 (0·0, 2·7)	0·0 (0·0, 0·4)	0·0 (0·0, 9·5)	0·0 (0·0, 1·4)
Mozambique							
Target: CHAMPS	2017-2021	448	8	1·8 (1·2, 3·4)	1·7 (1·3, 2·4)	26·3 (17·5, 49·7)	24·8 (19·0, 35·1)
Target: Catchment(s)	2017-2021	288	4	1·4 (0·9, 3·5)	1·1 (0·7, 3·1)	20·2 (13·0, 50·5)	15·9 (10·1, 44·7)
Sierra Leone	2019-2021	339	4	1·2 (0·8, 3·0)	1·2 (0·8, 2·4)	11·0 (7·4, 27·6)	11·0 (7·4, 22·1)
South Africa	2017-2021	804	7	0·7 (0·6, 1·7)	0·7 (0·5, 1·2)	18·5 (12·3, 34·9)	14·4 (10·3, 24·6)

Supplemental Table 10. Characteristics of CHAMPS cases consented for MITS and with neural tube defects anywhere in the mortality causal chain, by site.

	Bangladesh	Ethiopia	Kenya	Mali	Mozambique	Sierra Leone	South Africa	Total
	2017-2021	2019-2021	2017-2021	2017-2021	2017-2021	2018-2021	2017-2021	2017-2021
	MITS = 309	MITS = 311	MITS = 560	MITS = 208	MITS = 583	MITS = 416	MITS = 845	MITS = 3232
	NTD[1] = 4	NTD[1] = 44	NTD[1] = 1	NTD[1] = 0	NTD[1] = 9	NTD[1] = 4	NTD[1] = 7	NTD[1] = 69
	n (%)[2]	n (%)[2]	n (%)[2]	n (%)[2]	n (%)[2]	n (%)[2]	n (%)[2]	n (%)[2]
**Case characteristics**								
**Age group[3]**								
Stillbirths	2 (50·0)	38 (86·4)	0 (0·0)	0 (0·0)	6 (66·7)	3 (75·0)	2 (28·6)	51 (73·9)
Deaths <24 hours	1 (25·0)	4 (9·0)	0 (0·0)	0 (0·0)	2 (22·2)	1 (25·0)	1 (14·3)	9 (13·0)
Early neonates, 1-6 days	0 (0·0)	0 (0·0)	1 (100·0)	0 (0·0)	1 (11·1)	0 (0·0)	2 (28·6)	4 (5·8)
Late neonates, 7-27 days	1 (25·0)	1 (2·3)	0 (0·0)	0 (0·0)	0 (0·0)	0 (0·0)	1 (14·3)	3 (4·4)
Infants, 28-364 days	0 (0·0)	1 (2·3)	0 (0·0)	0 (0·0)	0 (0·0)	0 (0·0)	1 (14·3)	2 (2·9)
Children, 1-5 years	0 (0·0)	0 (0·0)	0 (0·0)	0 (0·0)	0 (0·0)	0 (0·0)	0 (0·0)	0 (0·0)
**Sex**								
Male	2 (50·0)	9 (20·5)	0 (0·0)	0 (0·0)	2 (22·2)	0 (0·0)	2 (28·6)	15 (21·7)
Female	2 (50·0)	35 (79·6)	1 (100·0)	0 (0·0)	7 (77·8)	4 (100·0)	5 (71·4)	54 (78·3)
**Location of death**								
Community	0 (0·0)	1 (2·3)	0 (0·0)	0 (0·0)	0 (0·0)	0 (0·0)	0 (0·0)	1 (1·5)
Facility	4 (100·0)	43 (97·7)	1 (100·0)	0 (0·0)	9 (100·0)	4 (100·0)	7 (100·0)	68 (98·6)
**Year of death**								
2017	0 (0·0)	0 (0·0)	0 (0·0)	0 (0·0)	0 (0·0)	0 (0·0)	3 (42·9)	3 (4·4)
2018	1 (25·0)	0 (0·0)	0 (0·0)	0 (0·0)	0 (0·0)	0 (0·0)	1 (14·3)	2 (2·9)
2019	1 (25·0)	4 (9·0)	1 (100·0)	0 (0·0)	6 (66·7)	2 (50·0)	2 (28·6)	16 (23·2)
2020	1 (25·0)	15 (34·1)	0 (0·0)	0 (0·0)	2 (22·2)	1 (25·0)	0 (0·0)	19 (27·5)
2021	1 (25·0)	25 (56·8)	0 (0·0)	0 (0·0)	1 (11·1)	1 (25·0)	1 (14·3)	29 (42·0)
**Season of death[4]**								
Dry	4 (100·0)	35 (79·6)	0 (0·0)	0 (0·0)	5 (55·6)	4 (100·0)	5 (71·4)	53 (76·8)
Rainy	0 (0·0)	9 (20·5)	1 (100·0)	0 (0·0)	4 (44·4)	0 (0·0)	2 (28·6)	16 (23·2)
**VA CoD[5,6]**								
Infection	0 (0·0)	0 (0·0)	0 (0·0)	0 (0·0)	0 (0·0)	0 (0·0)	0 (0·0)	0 (0·0)
Trauma	0 (0·0)	0 (0·0)	0 (0·0)	0 (0·0)	0 (0·0)	0 (0·0)	0 (0·0)	0 (0·0)
Other	4 (100·0)	44 (100·0)	1 (100·0)	0 (0·0)	6 (100·0)	4 (100·0)	5 (100·0)	64 (100·0)
**Maternal characteristics[7]**								
**Age at delivery[6]**								
<20 years	0 (0·0)	5 (16·7)	0 (0·0)	0 (0·0)	1 (12·5)	0 (0·0)	0 (0·0)	6 (12·5)
20-24 years	2 (66·7)	7 (23·3)	0 (0·0)	0 (0·0)	2 (25·0)	2 (50·0)	1 (50·0)	14 (29·2)
25-29 years	1 (33·3)	9 (30·0)	0 (0·0)	0 (0·0)	2 (25·0)	1 (25·0)	1 (50·0)	14 (29·2)
≥30 years	0 (0·0)	9 (30·0)	1 (100·0)	0 (0·0)	3 (37·5)	1 (25·0)	0 (0·0)	14 (29·2)
**Affiliated religion[6]**								
Christian	0 (0·0)	6 (14·6)	1 (100·0)	0 (0·0)	5 (83·3)	2 (50·0)	3 (100·0)	17 (28·8)
Hindu	1 (25·0)	0 (0·0)	0 (0·0)	0 (0·0)	0 (0·0)	0 (0·0)	0 (0·0)	1 (1·7)
Muslim	3 (75·0)	35 (85·4)	0 (0·0)	0 (0·0)	1 (16·7)	2 (0·0)	0 (0·0)	41 (69·5)
Other	0 (0·0)	0 (0·0)	0 (0·0)	0 (0·0)	0 (0·0)	0 (0·0)	0 (0·0)	0 (0·0)
**Education[6]**								
None	0 (0·0)	1 (100·0)	0 (0·0)	0 (0·0)	1 (33·3)	0 (0·0)	0 (0·0)	2 (28·6)
Primary	1 (50·0)	0 (0·0)	0 (0·0)	0 (0·0)	0 (0·0)	0 (0·0)	0 (0·0)	1 (14·3)
Secondary	1 (50·0)	0 (0·0)	0 (0·0)	0 (0·0)	1 (33·3)	0 (0·0)	0 (0·0)	2 (28·6)
Tertiary	0 (0·0)	0 (0·0)	0 (0·0)	0 (0·0)	1 (33·3)	0 (0·0)	1 (100·0)	2 (28·6)
**Alcohol during pregnancy[6]**								
Yes	0 (0·0)	0 (0·0)	0 (0·0)	0 (0·0)	0 (0·0)	0 (0·0)	0 (0·0)	0 (0·0)
No	3 (100·0)	5 (100·0)	0 (0·0)	0 (0·0)	2 (100·0)	4 (100·0)	2 (100·0)	16 (100·0)
**Smoking during pregnancy[6]**								
Yes	0 (0·0)	0 (0·0)	0 (0·0)	0 (0·0)	0 (0·0)	0 (0·0)	0 (0·0)	0 (0·0)
No	3 (100·0)	5 (100·0)	0 (0·0)	0 (0·0)	2 (100·0)	4 (100·0)	2 (100·0)	16 (100·0)
**Number of ANC visits[6]**								
0	0 (0·0)	10 (62·5)	0 (0·0)	0 (0·0)	0 (0·0)	0 (0·0)	0 (0·0)	10 (31·3)
1-2	2 (66·7)	3 (18·8)	0 (0·0)	0 (0·0)	0 (0·0)	0 (0·0)	2 (100·0)	7 (21·9)
3-4	1 (33·3)	1 (6·3)	0 (0·0)	0 (0·0)	5 (71·4)	2 (66·7)	0 (0·0)	9 (28·1)
5-6	0 (0·0)	2 (12·5)	0 (0·0)	0 (0·0)	1 (14·3)	1 (33·3)	0 (0·0)	4 (12·5)
7-8	0 (0·0)	0 (0·0)	0 (0·0)	0 (0·0)	1 (14·3)	0 (0·0)	0 (0·0)	1 (3·1)
9-10	0 (0·0)	0 (0·0)	1 (100·0)	0 (0·0)	0 (0·0)	0 (0·0)	0 (0·0)	1 (3·1)

ANC, antenatal clinic; BD, Bangladesh; CBD, congenital birth defects; CHAMPS, Child Health and Mortality Prevention Surveillance Network; CoD; cause of death; ET, Ethiopia; ICD-10, International Classification of Diseases, Revision 10; KE, Kenya; MITS, minimally invasive tissue sampling; ML, Mali; MZ, Mozambique; NTD, neural tube defects; SL, Sierra Leone; VA, verbal autopsy; ZA, South Africa.

[1]NTD, ICD-10 codes Q00.0, Q00.1, Q00.2, Q01.0-Q01.9, Q05.0-Q05.9.

[2]Percentages (column distributions) may not sum to 100% due to rounding.

[3]Stillbirths [no spontaneous breathing or movement at time of delivery and (1) weighing >1 kg and/or (2) estimated gestational age ≥28 weeks)]; neonates (1-28 days); infants (29-364 days); children (1-5 years).

[4]Dry, rainy: BD (November – May, June – October); ET (October – May, June – September); KE (July & December – March, April – June & August – November); ML (November – May, June – October); MZ (May – October, November – April); SL (October – May, June – September); ZA (March – November, December – February).

[5]Inter-VA algorithm: Infection (ICD-10 codes 01, 10.3-10.5); trauma (ICD-10 code 12).

[6]Missing: VA CoD (MZ, n=3; ZA, n=2); age at delivery (BD, n=1; ET, n=14; MZ, n=1; ZA, n=5); religion (ET, n=3; MZ, n=3; ZA, n=4); education (BD, n=2; ET, n=43; KE, n=1; MZ, n=6; SL, n=4; ZA, n=6); alcohol consumption during pregnancy (BD, n=1; ET, n=39; KE, n=1; MZ, n=7; ZA, n=5); smoking during pregnancy (BD, n=1; ET, n=39; KE, n=1; MZ, n=7; ZA, n=5); number of ANC visits (BD, n=1; ET, n=28; MZ, n=2; SL, n=1; ZA, n=5).

[7]All characteristics pertinent to time of pregnancy.

Supplemental Table 11. Frequency of co-occurring causes of death among CHAMPS cases consented for MITS and with NTDs anywhere in the mortality causal chain (2017-2021).

Co-occurring cause of death	Bangladesh	Ethiopia	Kenya	Mali	Mozambique	Sierra Leone	South Africa	Total
Perinatal asphyxia/hypoxia[2-3]	2	10	0	0	0	2	0	14
Other congenital birth defects[2-3]	1	2	0	0	0	0	0	3
Sepsis[2-3]	1	4	0	0	0	0	2	7
Congenital infections[2-3]	0	2	0	0	0	0	0	2
Lower respiratory infections[2-3]	0	1	0	0	0	0	2	3
Meningitis/encephalitis[2-3]	0	2	0	0	0	0	1	3
None – only NTD	2	31	1	0	9	2	4	49
Total	6	52	1	0	9	4	9	81

CHAMPS, Child Health and Mortality Prevention Surveillance Network; GBD, global burden of disease; ICD-10, International Classification of Diseases, Revision 10; IHME, Institute for Health Metrics and Evaluation; MITS, minimally invasive tissue sampling; NTD, neural tube defects. [1]Baliankandi, Bangladesh

(2017-2021); Harar and Kersa (2019-2021), and Haramaya (2020-2021), Ethiopia;
Kisumu, Kenya (2017-2021); Bamako, Mali (2017-2021);
Manhica and Quelimane, Mozambique (2017-2021);
Makeni, Sierra Leone (2018-2021); Soweto, South Africa (2017-2021).
[2]ICD-10 codes categorized using IHME GBD categories.
[3]Includes immediate, underlying, and comorbid causes of death 1-8.
NTD ICD-10 codes: anencephaly (Q00.0), craniorachischisis (Q00.1),
iniencephaly (Q00.2), encephalocele (Q01.0-Q01.9), spina bifida (Q05.0-Q05.9).

Supplemental Table 12. Folic acid fortification policy and fortification characteristics in CHAMPS sites.

Site	Folic acid fortification policy, fortification food vehicle, and nutrient levels in fortification standard[1]	Fortification program performance[1,2]
Bangladesh	Voluntary fortification – Rice (1·7 mg/kg)	Rice that is fortified = 2%
	Voluntary fortification – Wheat flour (2 mg/kg)	Wheat flour that is fortified = 0%
Ethiopia	Voluntary fortification – Wheat flour (2 mg/kg)	Wheat flour that is fortified = 0%
Kenya	Mandatory fortification – Maize flour (1·5 mg/kg)	Wheat flour that is fortified = 35%
	Mandatory fortification – Wheat flour (1·5 mg/kg)	Maize flour that is fortified = 28%
Mali	Mandatory fortification – Wheat flour (2·5 mg/kg)	Wheat flour that is fortified = 90%
Mozambique	Mandatory fortification – Maize flour (2 mg/kg)	Maize flour that is fortified = 70%
	Mandatory fortification – Wheat flour (2 mg/kg)	Wheat flour that is fortified = 60%
Sierra Leone	Voluntary fortification – Wheat flour (2·08 mg/kg)	Wheat flour that is fortified = 90%
South Africa	Mandatory fortification – Wheat flour (1·43 mg/kg)	Wheat flour that is fortified = 90%
	Mandatory fortification – Maize flour (2 mg/kg)	Maize flour that is fortified = 50%

[1]Global Fortification Data Exchange. Dashboard: Country Fortification. Accessed 13 Feb 2022. [http://www.fortificationdata.org].
[2]Proportion of fortification food vehicle in the country that is fortified.

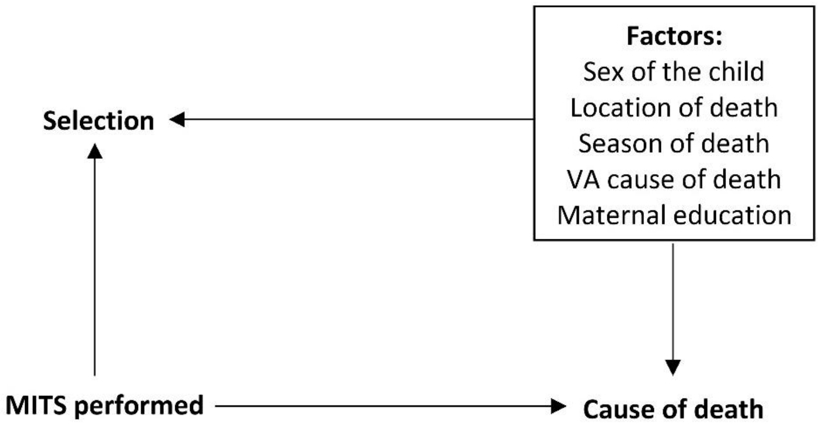

Supplemental Figure 1. Directed acyclic graph depicting the relationship between MITS performed and cause of death among CHAMPS cases. Estimates are susceptible to selection bias as all analysis are restricted to cases for whom MITS were performed. This biasing path may be closed by adjusting for factors known to affect both MITS consent and cause of death. CHAMPS, Child Health and Mortality Prevention Surveillance Network; MITS, minimally invasive tissue sampling; VA, verbal autopsy.

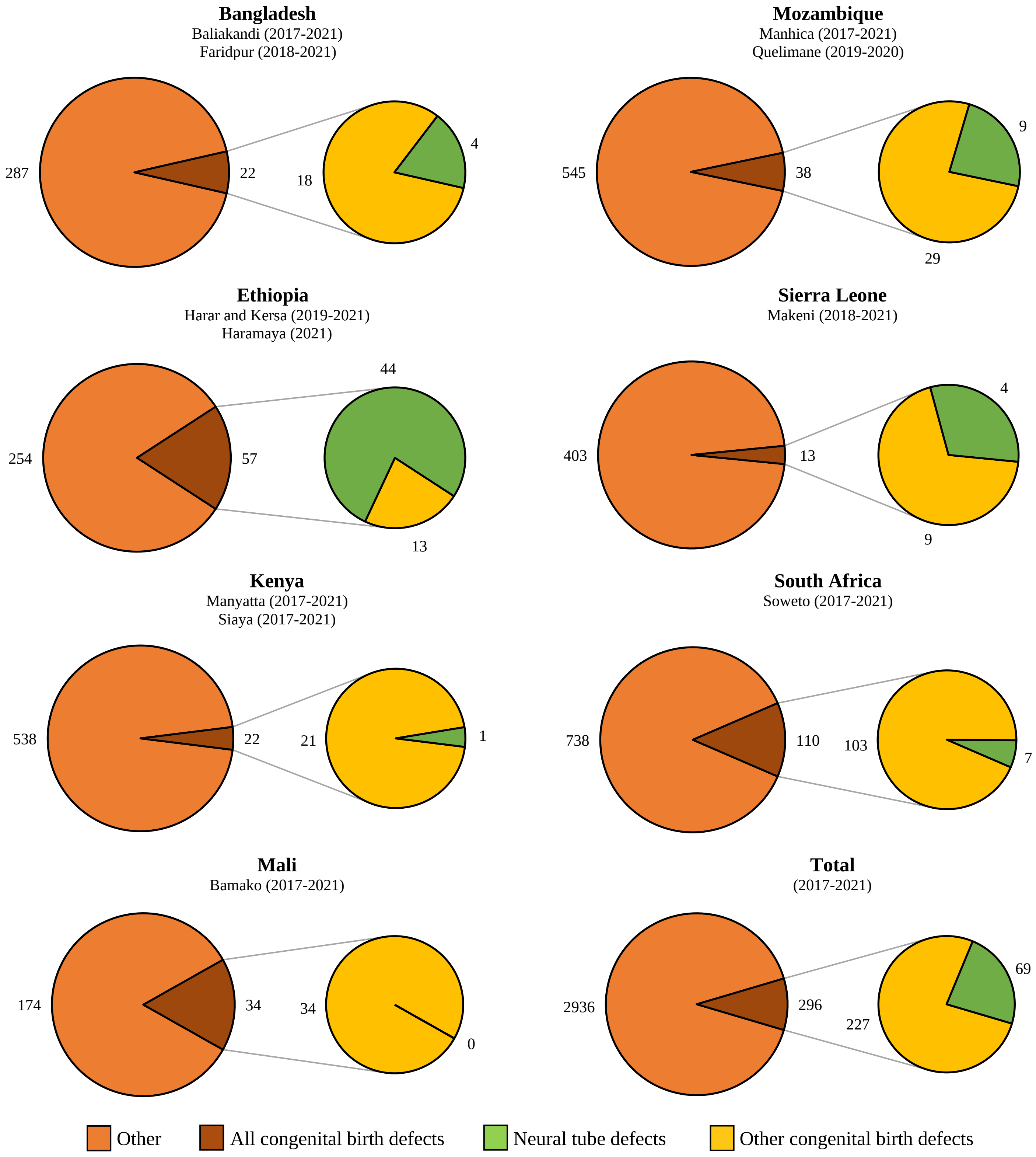

Supplemental Figure 2. Frequency of fatal congenital birth defects and neural tube defects anywhere in the causal chain among all CHAMPS cases consented for MITS by site. CHAMPS, Child Health and Mortality Prevention Surveillance Network; ICD-10, International Classification of Diseases, Revision 10; MITS, minimally invasive tissue sampling. Congenital birth defects, ICD-10 codes G71, G80, Q00-Q99; neural tube defects, ICD-10 codes Q00-Q00.0, Q00.1, Q00.2, Q01-Q01.9, Q05-Q05.9. Includes immediate, underlying, and comorbid causes of death 1-8.
